# 

**Published:** 1873-03

**Authors:** 


### TABLE OF CONTENTS.

ORIGINAL COMMUNICATIONS.

Chronic Diarrhœa as it Occurred During and Since the Late War. By Alban S. Payne, M.D., . . . . . . . . . . . . . . . 129
The Improved Galvano-Puncture Needles. By Alex. Murray, M.D., . . 145

SELECTIONS.

Chlorate of Potassa in Scrofulous Cases. By C. A. Clarkson, M.D., . . 157
Oleum Terebinthinæ in Acute Affections of the Middle Ear. By Fredr. Eugene Weber, . . . . . . . . . . . . . . . 160
Ophthalmology and Otology. By C. E. Wright, M.D., . . . . . . 161
Neuralgia of the Testicle, . . . . . . . . . . . . . 163

EXTRACTS AND GLEANINGS.

A Cure for Corns—Puerperal Convulsions, 165; Aneurism of Arch of Aorta—Treatment of Catarrh of the Lachrymal Sac—Sciatica, 166; Asthma—Rheumatism; Treatment of Uterine Hemorrhage by Sulphate of Quinine, 167; Galvano-Emesis—Arrest of the Destruction of Lung Tissue in Chronic Phthisis by Means of the Inhalation of Oxygenated Essences—Pruritus of the Meatus, 168; The Treatment of Infantile Paralysis—Calomel in Diseases of the Eye—Epilepsy, 169; Epithelioma Cured by Creosote—Carbolic Acid Internally—Neuralgia, Galvanism, 170; Insanity in Children—Muriate of Opium—Night Sweats, 171; Coal Oil as a Derivative in Congestion—Alcoholic Paraplegia—Cysts in the Liver in Childhood—Substitute for Granville's Lotion, 172; Subcutaneous Injection of Morphia—Inhalation of Bromine—Caffeine—Fluid Extract of Compound Powder of Jalap and Senna, 173; Puerperal Convulsions—Typhoid Fever—Lacto-Phosphate of Lime in Typhoid Fever—Veratrum Viride with Morphia in Pericarditis, 174; Hypodermic Use of Ergot—Vaccination from a Re-vaccinated Person; Fluid Extract of Aconite as a Local Application—Gonorrhœa, Carbolic Acid, 175; Tapeworm—Abdominal Aneurism Cured by Aortic Compression—"Open-Air" Treatment of Hooping-Cough—Chloride of Zinc and Glycerin in Ophthalmia of New-Born Children, 176; Deodorized Tincture of Iodine—To Kill Lice—The Cure of Scurvy, 177; Chloracetic Acid for Warts—Disinfection of Rooms—Ginger Wine—Itch Ointment, 178; Warm Bath in Insanity and in Burns—Gelseminum in Urethro-Cystitis—Elixir of Pepsin and Bismuth—Bisulphide of Carbon, 179; Carbolic Acid in Skin Diseases—For Chapped Hands and Lips—Creosote in Anthrax—Phosphorus Pills, 180; Bodily Quietude in the Diseases of Children—Alcohol and Renal Disease—Ulcerative Stomatitis, 181; Constipation Relieved by Electricity—Mixed Vapors for Anæsthesia—Erethema or Simple Inflammation of the Skin—Eczema, 182; Measles and Pneumonia—Treatment of Constitutional Syphilis in Infants—Happy Combination for Chronic Diarrhœa—Glycerin Lemonade in Diabetes Mellitus, 183; Chloral and its Administration—Opium in Wounds of the Intestines—Poisonous Effects of Zinc Utensils—For Hooping-Cough, 184; Reduction of Paraphymosis—Senna Coffee—Ingrowing Toe-Nail—Cimicifuga Racemosa in Small Pox, 185.

EDITORIAL AND MISCELLANEOUS.

The Georgia Medical Association; A Hint to Subscribers, . . . 186
Communications; Please Take Notice; Appleton's Journal of Literature, Science and Art; Lippincott's Magazine, an Illustrated Monthly of Popular Literature and Science; The London Lancet, American Reprint, . . 187
Archives of Scientific and Practical Medicine; Braithwaite's Retrospect and Half-Yearly Abstract; Kind Words and Suggestions, . . . 188
List of Payments Received for the RECORD, Commencing with the January Number, . . . . . . . . . . . . . . 190
List of Payments, 1872; Our Friends, . . . . . . . . 191

